# The *PLOS Neglected Tropical Diseases* decade

**DOI:** 10.1371/journal.pntd.0005479

**Published:** 2017-04-20

**Authors:** Peter Hotez, Donald A. P. Bundy

**Affiliations:** 1 Sabin Vaccine Institute and Texas Children’s Hospital Center for Vaccine Development, National School of Tropical Medicine at Baylor College of Medicine, Houston, Texas, United States of America; 2 Department of Biology, Baylor University, Waco, Texas, United States of America; 3 Center for Health and Biosciences, James A Baker III Institute for Public Policy, Rice University, Houston, Texas, United States of America; 4 Scowcroft Institute of International Affairs, Bush School of Government and Public Service, College Station, Texas, United States of America; 5 Bill & Melinda Gates Foundation, Seattle, Washington, United States of America, and London, United Kingdom; Yale School of Public Health, UNITED STATES

The founding of *PLOS Neglected Tropical Diseases* (*PLOS NTDs*) a decade ago was intimately tied to the beginnings of a pro-poor and anti-poverty initiative in the aftermath of the Millennium Development Goals launch, together with advocacy by prominent global leaders and historic commitments from the Bill & Melinda Gates Foundation. The year 2005 was an important one for the journal and for NTDs. In March, the British Government released their report on the Commission for Africa [1], which served as a blueprint for a landmark group of eight (G8) Summit in Gleneagles focused on African development and the importance of parasitic diseases that went beyond malaria. In parallel two connected papers published in *PLOS Medicine*—one of the first high impact open access journals devoted to global health—looked at the bundling of an important group of African parasitic infections branded as “NTDs” and targets for integrated mass drug administration [2], possibly linked to the control of HIV/AIDS and malaria [3]. Those papers built on the foundations of “the great neglected diseases” as reflected in the first edition of *Disease Control Priorities* [4], and on the hard work of a generation of biomedical heroes committed to deworming for soil-transmitted helminth infections and schistosomiasis, as well as mass drug administration for lymphatic filariasis, onchocerciasis, and trachoma [5].

Such documents set the stage for a 2005 NTD summit in Berlin organized jointly by the World Health Organization (WHO) and the Gesellschaft für Technische Zusammenarbeit (GTZ, German Technical Cooperation Agency). Side meetings during the Berlin summit led to discussions with representatives from PLOS and the Bill & Melinda Gates Foundation regarding the importance of making data and content freely available to the community of scientists, public health experts, and global advocates committed to the world’s poverty-related diseases. The concept was born of a journal that would not only represent the needs of a community of scholars, but one which would simultaneously become a capacity building tool for disease experts living and working in Africa and in other disease-endemic regions of the world. It was recognized that providing free and open access to papers on NTDs to anyone with internet capabilities would be particularly empowering for those working in resource-poor circumstances. It could also give a voice to a community of scholars who might otherwise not submit papers to an international journal. From the very beginning, the *PLOS NTDs* editors, especially Dr. Serap Aksoy (who became Co-Editor-in-Chief), led writing workshops for African scientists, and eventually for scientists worldwide.

As a result of a catalytic investment from the Bill & Melinda Gates Foundation, the journal quickly got underway, and opened with a high level of interest and support from the founding leadership of PLOS, including visionaries such as Harold Varmus, Pat Brown, and Mike Eisen, among others [6]. The launch benefitted particularly from a very welcome, and deeply appreciated, early note of endorsement from *The Lancet* [7]. That generous act set a very nice standard of collegiality, and established a special relationship among the editors of *PLOS NTDs* and *The Lancet* family of journals, which has continued with subsequent open access journals devoted to parasitic and neglected diseases, such as *Parasites & Vectors*.

For the first and only print edition of *PLOS NTDs* the artist Emma Burns donated her painting, “A Ray of Hope”, which graced the inaugural issue in 2007 (Fig 1). The painting was then repurposed for the first edition of the book, *Forgotten People Forgotten Diseases* [8].

**Fig 1:**
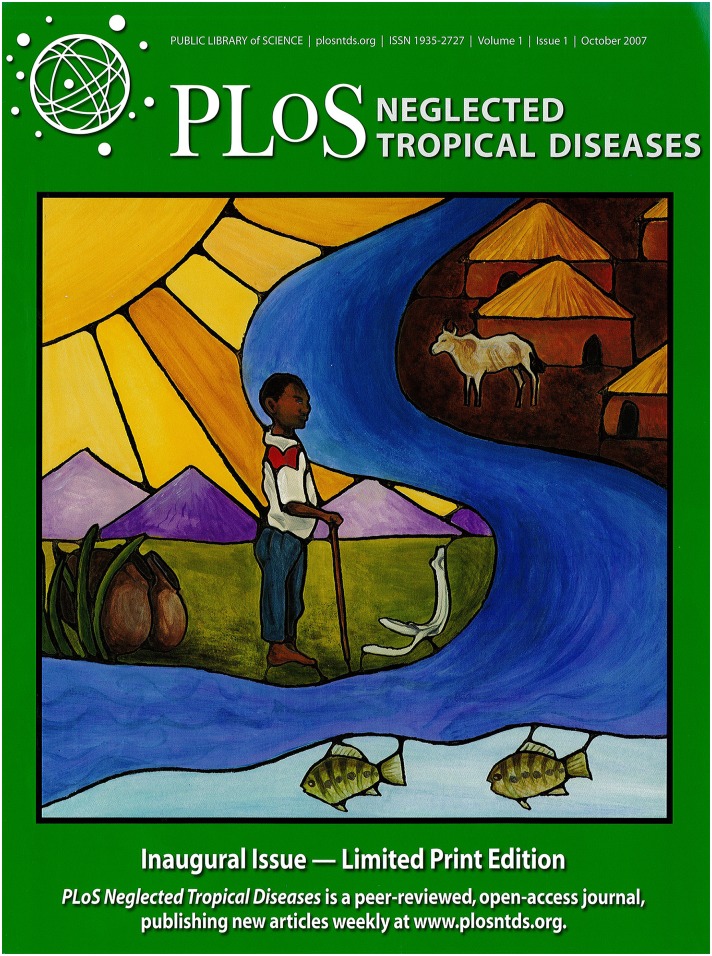
2007 inaugural issue of *PLOS NTDs*. Picture by Ms. Emma Burns, “A Ray of Hope”.

## Science capacity building at *PLOS NTDs*

Over the ensuing decade the capacity-building function of *PLOS NTDs* has remained paramount. To date, *PLOS NTDs* has published 4702 articles (research articles and front matter), written by a total of 8391 unique authors. Table 1 shows a geographical breakdown of the *PLOS NTDs* authors by continent, indicating an encouragingly high representation of authors from the “global south”–Africa, Asia, South America, and Oceania.

**Table 1:** Breakdown of the unique *PLOS NTDs* authors by continent group (information collected and provided by Charlotte Bhaskar at PLOS). (*Percentages add up to >100 because an article can have authors from more than one country).

Continent Group	#Unique Authors (DOIs)	Percentage of all Authors* (DOIs)
Africa	1,169	25%
Asia	1,262	27%
South and Central America	1,070	23%
Eastern Europe	60	01%
Oceania	437	09%
North America	2,062	44%
Western Europe	2,330	50%

**Fig 2:**
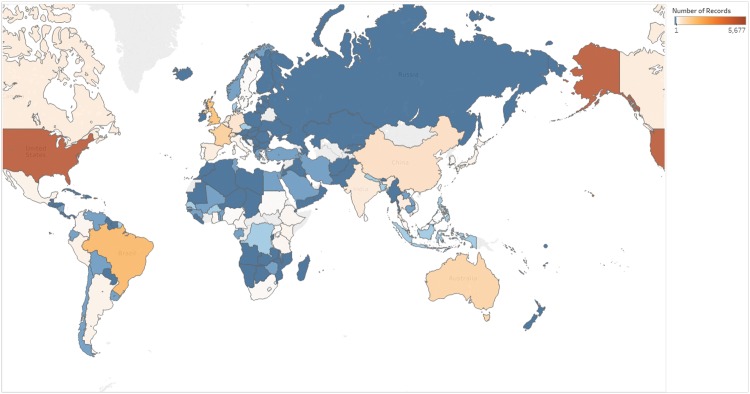
Author distribution of PLOS NTDs articles. Courtesy of Charlotte Bhaskar.

One special feature of PLOS NTD, as shown in Fig 2, is that there is a particularly high representation by authors from the large upper-middle income nations, including Brazil, China, India, Mexico, and several Southeast Asian nations, a powerful group whose voice has strengthened in parallel with the development of the journal. We would like to see more authors from Africa, the geographical location of a significant NTD burden, but note with appreciation that physicians and scientists from most of the sub-Saharan countries have chosen to author *PLOS NTDs* articles.

We believe that the strong authorship from outside the global “north” was not achieved by chance and *PLOS NTDs* has sought and maintained an unusually high level of representation of its editorial board members from disease-endemic countries. As shown in Table 2, among the more than 200 associate editors of *PLOS NTDs*, approximately 40 percent come from disease-endemic countries of Africa, Asia, and Latin America. One-third of the associate editors are women.

**Table 2:** Geographic origin of the *PLOS NTDs* associate editors (information collected and provided by Charlotte Bhaskar at PLOS).

Continent	Male	Female	Country total	Country percentage (%)
Africa	14	4	18	8.5
Asia	16	8	24	11.3
Europe	26	16	42	19.7
North America	56	29	85	39.9
South and Central America	21	10	31	14.5
Oceania	11	2	13	6.1
Gender total	144	69		
Gender percentage (%)	67.6	32.4		
Total from Africa, Asia, South America, Oceania			86	40.4
TOTAL AEs:	213			

Beyond these demographics, to date the editors of PLOS NTDs have hosted 24 manuscript-writing workshops in disease endemic countries [9].

## Press coverage and impact

The papers at *PLOS NTDs* have been highly cited: the journal has consistently ranked first or second of all tropical disease journals in terms of impact factor. We are confident in the credibility of this high citation rate as PLOS NTD was the first journal to use article-specific metrics to assess the audience of a given research paper, so that for each *PLOS NTDs* article it is now possible to see the number of times a paper has been viewed, downloaded or shared on social media. Our top paper to date has been viewed over 230,000 times and shared on social media more than one thousand times. Moreover, because we are an open access journal our articles are easily searched and seen by science and health journalists. Reports of *PLOS NTDs* papers have appeared in all of the major media outlets including *New York Times*, BBC News, *Guardian*, and others. During the 2014 Ebola outbreak in West Africa, and the 2015–16 Zika virus outbreak in South America, and now the 2016–17 yellow fever epidemic in Africa and Brazil, *PLOS NTDs* has been at the forefront in rapid publication and dissemination of critical information needed to fight these epidemics. We are already getting ready for what might be in store for 2017 and 2018.

## Concluding statement

We wanted to use this tenth anniversary to thank the readers, contributors, editors, and editorial staff of *PLOS NTDs*. We are especially proud of the high representation of authors and journal editors living and working in the disease endemic countries, and also the women scientists and disease experts serving as *PLOS NTDs* Co-Editor-in-Chief, and deputy and associate editors.

As with so many other initiatives launched in the “decade of global health” between 2000 and 2010, the Bill & Melinda Gates Foundation was instrumental in providing initial catalytic support for *PLOS NTDs*. On behalf of all those who have read or contributed to PLOS NTD we express heartfelt gratitude to Bill & Melinda Gates for the initial vision and commitment that made this journal possible.
